# Prioritizing Type of Industry through Health Risk Assessment of Occupational Exposure to Dimethylformamide in the Workplace

**DOI:** 10.3390/ijerph15030503

**Published:** 2018-03-13

**Authors:** Junghyun Lee, Miran Hahm, Da-An Huh, Sang-Hoon Byeon

**Affiliations:** Department of Health Science, Graduate School, Korea University, Seoul, 02841, Korea; ltl8368@hanmail.net (J.L.); miran5072@naver.com (M.H.); black1388@hanmail.net (D.-A.H.)

**Keywords:** dimethylformamide, risk assessment, occupational hazard, manufacturing industries

## Abstract

The purpose of this study was to classify hazards at an industrial level and evaluate the exposure risks of workers exposed to dimethylformamide (DMF) used as a solvent in the workplace and to determine industries that need priority measures in managing DMF exposure. We calculated hazard quotients at an industrial level. The exposure data of DMF in the workplace were obtained from the work environment monitoring program provided by the Korea Occupational Safety and Health Agency. The evaluation was conducted on textile manufacturing, leather manufacturing, chemical manufacturing, pharmaceutical manufacturing, and rubber manufacturing industries, which have many unit work sites handling DMF. The highest central tendency exposure and reasonable maximum exposure were 2.13 and 18.66 mg/m^3^ for the rubber product manufacturing industry, respectively. A total of 63.8% of workplaces in the textile manufacturing sector had a hazard quotient higher than 1. The highest risk for exposure to DMF is in the rubber and plastic manufacturing industry, and the lowest risk was in the medical materials and pharmaceutical manufacturing sector. Based on this study, effective management of DMF exposure could be achieved by establishing priority management measures for the textile and rubber and plastic product industries.

## 1. Introduction

Dimethylformamide (DMF) is a colorless and water-soluble liquid used as a solvent or additive in chemical industries. It is mainly used as a solvent in artificial leather production, textile coating processing, and urethane and acrylic fiber spinning [1]. It is also often used as an auxiliary solvent or booster of protective coatings, adhesives, films, and printing inks and is also used as a component of paint removers [2]. DMF is absorbed into the human body through the respiratory system, skin, and digestive organs, causing damage to the liver. Nausea, vomiting, diarrhea, abdominal pain, edema, malaise, jaundice, and nasal skin symptoms might develop due to the effects of DMF absorbed in the digestive system [3,4,5]. Because of its detrimental effect on human health, the Ministry of Employment and Labor (MOEL) in Korea and American Conference of Governmental Industrial Hygienists (ACGIH) had set the occupational exposure limit (OEL) for DMF at 30 mg/m^3^ [6,7,8,9]. In 2014, a total of 84,944 tons of DMF was used in Korea, which is 92% higher than in 1993. The number of exposed workers was approximately 3500, more than triple of that in 1993 [10,11]. In the United States, DMF is designated as a high-production volume chemical, with over 100,000 workers exposed to DMF. In Korea, more than 20 cases of occupational diseases have been caused by DMF after the first case reported in 1993 [12,13,14,15,16,17]. In Korea, DMF is designated as the target substance of the workplace environmental monitoring program (WEMP) to manage DMF exposure. It is mandatory to measure its concentration in the workplace more than once every six months in all workplaces handling DMF. The WEMP aimed to control the workplaces where the substance is handled, and they collect data on the type of work, main product, department name, process code, process name, unit workplace, handling time of hazard substances, and exposure concentration, among others.

The purpose of this program is to identify whether the regulatory thresholds are exceeded and to control the employee exposure to hazardous substances. Since DMF causes acute toxicity, such as hepatitis and hepatic necrosis at the initial stage of exposure, the MOEL recently established a post-placement health checkup in which a special health checkup for DMF is conducted within one month after exposure. However, the OEL is set by considering social and economic conditions as well as the human health risk. Therefore, it is different from the concentration associated with substantial health effects. Some studies have reported adverse health effects even at concentrations below the currently established exposure limit (30 mg/m^3^) [18,19,20,21,22,23,24,25]. Therefore, it is important not only to compare the DMF exposure with the regulatory threshold, but also to evaluate and manage the risks to the health of the workers through a quantitative risk assessment. Also, it is economically ineffective to manage each industry with different levels of exposure by applying the same method.

In this study, the overall DMF exposure levels and health risks of workplaces in each industries was evaluated using the work environment measurement data. According to the result of the risk assessment, we prioritized which industries need to be managed most urgently.

## 2. Materials and Methods

A total of 3121 DMF measurements were made in the 2014 work environment data. The total measurement data are classified according to the industry code of the workplace following the Korean Standard Industrial Classification (KSIC 9th) [26]. Of the 178 categories classified, the 5 sectors with the largest number of workplaces handling DMF were selected for evaluation. As such, the target manufacturing industries evaluated for DMF handling were textiles, leather and related products, chemicals and chemical products, basic pharmaceutical products and pharmaceutical preparations, and rubber and plastic products.

The use of DMF was significantly lower in industries other than the abovementioned five industries. Exposure estimates for 2737 measurements out of a total of 3121 measurements of the five industries were below detection limits or not detected.

The risk assessment was carried out in accordance with Chemical Hazard and Risk Assessment Guidelines (KOSHA GUIDE W-6-2012) of the Korea Occupational Safety and Health Administration (KOSHA) [27]. Given the lack of toxicity data to confirm human carcinogenicity, the unit risk for carcinogenicity risk assessment could not be obtained. Therefore, in this study, only the non-carcinogenic toxicity of DMF was evaluated.

We adopted the point of departure (POD) of the study results that is considered to be the most reliable and appropriate for dose response assessment for dose response evaluation. Reference concentrations in the workplaces (RfC_work_) were calculated by applying a correction factor appropriate for the Korean workers’ situation to the toxicity value of the study results. The correction factor is based on the values provided by KOSHA, which is based on the values of the correction factors proposed by the U.S. Environmental Protection Agency (EPA); EU European Chemicals Agency; and Japan Ministry of Health, Labor and Welfare.

DMF exposure measurements at workplaces were based on the 2014 WEMP. The measurement values were classified for each industry type, and the geometric mean (GM) and the geometric standard deviation (GSD) were obtained. Monte Carlo simulation was performed using Oracle Crystal Ball 11.1 (USA) to obtain a probabilistic distribution of exposure variables. Assuming a lognormal distribution, we obtained a central tendency exposure (CTE) and a reasonable maximum exposure (RME).

Hazard quotient (HQ) was used to determine the health risk for non-carcinogens. HQ was calculated by dividing the measured DMF concentration with the RfC_work_ as (Equation (1)):

HQ = DMF concentration (mg/m^3^)/RfC_work_ (mg/m^3^) (1)

A value greater than 1 indicates a potential risk of adverse health effects. In this study, the risk assessment was performed using both CTE and RME values according to the exposure assessment results, and the HQ for each was calculated. In addition, HQ was calculated for all data, and the ratio of industry type with HQ of 1 or more was calculated. Regarding factors mentioned above, we prioritize industry types that most urgently need to be managed.

## 3. Results

### 3.1. Dose–Response Assessment

In this study, the lowest observed adverse effect level (LOAEL) value for non-carcinogenic toxicity of DMF tested by Cirla et al. [7] was used to calculate the RfC value. This value was used to determine RfC in the U.S. EPA’s Integrated Risk Information System, which included a change in objective measurements of mild liver dysfunction and serum enzymes and liver enlargement of 100 workers exposed to DMF for an average of five years. This study calculated the LOAEL by checking the changes in objective measurements of mild liver dysfunction and serum enzymes and liver enlargement of 100 workers exposed to DMF for an average of five years.

To evaluate the toxicity of inhalation, back extrapolation was required for correction. The calibration parameters for RfC_work_ were determined and are shown in Table 1.

The quantitative calibration parameters were applied at 0.5 considering the actual exposure time and the respiration rate between the worker and the non-worker. Having used the data from the epidemiological study of factory workers, we utilized 1 and 5 as the values for interspecies and intraspecies differences, respectively. As the study’s average duration was five years, we applied 1 as the uncertainty correction. Next, a value of 5 was applied as the severity correction factor. Finally, the inhalation RfC in the workplace (RfC_work_) for DMF was calculated as 0.44 mg/m^3^.

### 3.2. Exposure Assessment

The GM and GSD of the DMF exposure measurements, except for data that are not detected or below the detection limit, are shown in Table 2.

The industries with the highest average values of exposure were textile product manufacturing (4.92 mg/m^3^), rubber and plastic manufacturing (4.50 mg/m^3^), leather product processing (3.27 mg/m^3^), chemical product manufacturing (0.99 mg/m^3^), and basic chemical manufacturing (0.06 mg/m^3^).

As a result of the Monte Carlo simulation to obtain a stochastic distribution of these measurements, the CTE and RME of each industry type were 1.71 mg/m^3^ and 17.97 mg/m^3^ for textile products, 2.13 mg/m^3^ and 18.66 mg/m^3^ for rubber products, 0.45 mg/m^3^ and 13.86 mg/m^3^ for rubber products, and 0.00 mg/m^3^ and 7.08 mg/m^3^ for chemical product manufacturing and basic chemical manufacturing, respectively (Table 3).

Exposure assessment results showed high exposure levels in the textile and rubber and plastic manufacturing industries and low exposure levels in the medical materials and pharmaceutical manufacturing industries.

### 3.3. Risk Assessment

Using the CTE and RME obtained through exposure assessment, we calculated the HQ for each of the five industries (Table 4). For RME, HQ was more than 1 in all industries other than basic chemicals, and CTE exceeded 1 for HQ in textiles, leather products, rubber, and plastic product industries.

As a result of the HQ output, workers in the textile, leather, rubber, and plastic manufacturing industries were considered to generally have a significant health risk. Only the HQ of RME of the chemical manufacturing exceeded 1. Meanwhile, in the case of basic pharmaceutical product manufacturing, the HQ of CTE and RME was less than 1. However, in the worst case, all of them are likely to have a potential risk; thus, they will need to be managed continuously. The individual HQ values were obtained to identify the risk level of each industry type. For the rubber and plastic manufacturing industries, the CTE and RME values were 4.84 and 42.41, respectively.

To compare the risk level by industry, the ratio of workplaces with HQ exceeding 1 by industry was calculated (Table 4).

For the textile, rubber, and rubber and plastic products industries where the HQ for RME and CTE are all at least 1, 62.5% of the total workplace had a potential exposure to DMF. In addition, 24.7% of the chemical manufacturing sector and 8.2% of the basic pharmaceutical manufacturing industry had HQ values exceeding 1.

## 4. Discussion

The purpose of this study was to evaluate the overall risk of occupational exposure for workplaces handling DMF in Korea and to identify the differences in exposure and risk according to industry.

In this study, the WEMP data used are from the whole survey about the workplace handling the DMF; thus, many unit workplaces that deal with DMF but do not show any substantial exposure were included. Therefore, in the exposure assessment of this study, we tried to understand the overall risk of exposure in the workplace in which actual exposure occurs, except in workplaces where exposure was below the detection limit. However, because detailed information on the unit workplace on non-detectable data cannot be evaluated and non-detected data account for a large part in each industry, the risk index derived by using CTE and RME in this paper may be higher than the risk level of the whole workplace.

In dose–response assessment, the CTE was used to assess the overall exposure level of the workplace by industry, and the RME was used to assess the risks that would arise assuming the worst exposure conditions, which means that a risk assessment should be performed in a conservative approach [28].

In the risk determination, the result of risk using HQ is meaningful only to identify the overall risk of the workplace exposed to DMF. As such, all workplaces of four industries with HQ less than 1 cannot be identified as safe. This means that the HQs obtained for CTE and RME are inadequate to determine the individual risks for each site. As such, to accurately assess the health risks posed by chemicals in the workplace, each workplace should be assessed individually according to the conditions of the site.

In this study, individual HQs were obtained for each workplace exposure, and the ratio of workplaces with HQ exceeding 1 was determined to compare the risk level among industries. Considering the total number of workplaces in the industry, the rubber and plastic manufacturing industries were found to have the highest number of workplaces with a risk of exposure to DMF.

When considering the ratio of workplaces with an HQ higher than 1 in each industry, DMF exposure control was found to be the most urgent in the rubber and plastic product manufacturing and textile manufacturing industries.

Typical processes involved in the textile product manufacturing sector, which are expected to have high exposure levels, are usually in the following order: polymerization, spinning, drying, heating, winding, finishing, and packing [29]. In particular, spinning and winding are the processes during which worker exposure to DMF is the highest. The humidity of workplaces is high during spinning; the workers submerge their hands in the solution in the spinning bath to arrange the fiber, thus exposing the workers to DMF through the skin and respiratory system. Meanwhile, during winding, the workers must check the equipment frequently to determine the quality of fiber winding. During these checks, the workers can be exposed to the DMF present in the fiber. Before the winding process, the fiber is dried by heating it under high temperatures. Therefore, the diffusion of DMF vapor during the winding process is of special concern [30].

The rubber and plastic manufacturing industry, including synthetic leather, is ranked to have the second highest health risk from DMF exposure. Urethane resin, which is used as a coating material in the production of synthetic leather, contains 20–70% DMF. DMF is also used as a solvent in the manufacture of synthetic leather. Therefore, DMF exposure via the respiratory system is of concern in most synthetic leather manufacturing processes. In the printing process, DMF has been used as a solvent to dissolve the toning pigments. In this process, the color pigments need to be replaced often to match the color. As such, the possibility of exposure of workers to DMF is high during the process [17].

The current OEL of 30 mg/m^3^ in Korea is approximately 68 times higher than the RfC_work_ (0.44 mg/m^3^) of DMF obtained in this study. Human health hazards as well as social, economic, and technical requirements are considered in setting the OEL. Therefore, it is set higher than the RfC, which only considers the human health risks. However, the current OEL should be reassessed for the following reasons. In the case of Cirla et al. [7], the worker health risks appeared below the current OEL [4,31]. Considering these points, the current OEL is insufficient to fully protect against the health risks of DMF. Thus, the current OEL should be lowered and managed more strictly.

As the local exhaust system of each workplace and the work environment significantly influence the exposure level, measures should be taken to create a work environment with minimal exposure to DMF regardless of the industry. However, because modifying the entire workplace can be complicated and socially and economically costly, modifying the workplace based on the group requiring priority management can be effective [6,32].

The results of the risk assessment showed that DMF exposure should be urgently managed in rubber and plastic manufacturing industries and textile manufacturing industries. The overall DMF exposure can be managed efficiently by prioritizing the plastic product manufacturing and textile product manufacturing industries.

The WEMP data used in this study were only used to evaluate respiratory exposure, obtaining or evaluating the amount of exposure through skin absorption was not possible. Additional studies on DMF exposure through skin absorption are needed. Moreover, in this study, only the risk of exposure to DMF is evaluated, but workers are also exposed to many other hazardous chemicals in the workplace.

In particular, the human body is significantly affected when exposed to both toluene and DMF [33]. However, data on this exposure is lacking. Further, the interaction of DMF with other hazardous chemicals was not assessed and is a limitation of this study.

The results of this study showed that even at workplaces that do not exceed OEL, workers may still be exposed to DMF at concentrations above RfC. A total of 42.6% of the total workplaces have exposure levels above RfC. In particular, 63.8% and 62.5% of the textile and the rubber and plastic manufacturing industries, respectively, showed exposure levels above RfC. The exposure concentrations and risks of DMF should also be evaluated in Korea.

## 5. Conclusions

In this study, we suggested industries that should be prioritized for managing the health risk of DMF exposure by evaluating the overall risk of DMF in the workplaces using the WEMP data and by identifying the difference in DMF exposure among industries. The comparison of the measured concentrations and the results of the risk assessment showed that the textile and plastic product manufacturing industries had the highest risk for DMF exposure, while the risk DMF exposure is relatively low in the chemical manufacturing industry. Therefore, DMF exposure should be managed primarily in the textile and plastic product manufacturing industries.

## Figures and Tables

**Table 1:** Values used to calculate the RfC_work_ of DMF.

Steps	Categories		Values
POD *			LOAEL ** 22 mg/m^3^ (human, inhalation)
Route-to-route extrapolation	Dose scaling from animals to humans		1
Quantitative correction	NOAEL_ADJ_		6/8 × 5/5 × 0.83/1.25 = 0.5
	NOAEL_HEC_		1
Uncertainty correction	Interspecies		1
	Intraspecies		5
	Duration	≥6 months	1
	Severity	LOAEL **	5
RfC_work_	22 mg/m^3^ × 1 × 0.5 × 1/1 × 5 × 1 × 5 = 0.44 mg/m^3^		0.44 mg/m^3^

POD *, point of departure; LOAEL **, lowest observed adverse effect level; NOAEL, no observed adverse effect level; NOAEL_ADJ_, NOAEL adjusted for duration; NOAEL_HEC_, NOAEL adjusted to a human-equivalent concentration; RfC_work_, reference concentrations in the workplaces.

**Table 2:** Geometric mean and geometric standard deviation for the measurement values.

Type of Industry	N *	GM (mg/m^3^)	GSD	Range (mg/m^3^)
Textiles	450	4.92	9.54	0.00–7.87
Leather and related	337	3.27	4.86	0.00–28.94
Chemicals and related	987	0.99	2.73	0.02–9.29
Basic pharmaceutical	427	0.06	0.27	0.04–0.99
Rubber and plastic	536	4.50	6.06	0.01–12.49

N * The number of used data when calculating GM and GSD except for the values that are not detected or less than limit of detection. GM, geometric mean; GSD, geometric standard deviation.

**Table 3:** Results for CTE, RME, and HQ to dimethylformamide.

Results	A *	B *	C *	D *	E *
CTE (mg/m^3^)	1.71	0.45	0.00	0.00	2.13
RME (mg/m^3^)	17.97	13.86	7.08	0.18	18.66
HQ for CTE	3.89	1.02	0.00	0.00	4.84
HQ for RME	40.84	31.50	16.09	0.41	42.41

A *: textiles; B *: leather and related; C *: chemicals and related; D *: basic pharmaceutical; E *: rubber and plastic; CTE: central tendency exposure; RME: reasonable maximum exposure; HQ: hazard quotient.

**Table 4:** Number of workplaces with HQ exceeding 1.

Type of Industry	Total n *	n (HQ > 1) **	Percentage (%) ***
Textiles	450	287	63.8
Leather and related	337	207	61.4
Chemicals and related	987	244	24.7
Basic pharmaceutical	427	35	8.2
Rubber and plastic	536	335	62.5

*: The number of measurement data except for the values that are not detected or less than limit of detection. **: The number of measurement data with hazard quotient exceeding one. ***: The number of measurement data with hazard quotient exceeding one/the number of total data in each industry. HQ: Hazard quotient.
